# Optimal Universal Uncertainty Relations

**DOI:** 10.1038/srep35735

**Published:** 2016-10-24

**Authors:** Tao Li, Yunlong Xiao, Teng Ma, Shao-Ming Fei, Naihuan Jing, Xianqing Li-Jost, Zhi-Xi Wang

**Affiliations:** 1 School of Science, Beijing Technology and Business University, Beijing 100048, China; 2 School of Mathematics, South China University of Technology, Guangzhou, Guangdong 510640, China; 3 Max Planck Institute for Mathematics in the Sciences, Leipzig 04103, Germany; 4 State Key Laboratory of Low-Dimensional Quantum Physics and Department of Physics, Tsinghua University, Beijing 100084, China; 5 School of Mathematical Sciences, Capital Normal University, Beijing 100048, China; 6 Department of Mathematics, North Carolina State University, Raleigh, NC 27695, USA

## Abstract

We study universal uncertainty relations and present a method called joint probability distribution diagram to improve the majorization bounds constructed independently in [Phys. Rev. Lett. **111**, 230401 (2013)] and [J. Phys. A. **46**, 272002 (2013)]. The results give rise to state independent uncertainty relations satisfied by any nonnegative Schur-concave functions. On the other hand, a remarkable recent result of entropic uncertainty relation is the direct-sum majorization relation. In this paper, we illustrate our bounds by showing how they provide a complement to that in [Phys. Rev. A. **89**, 052115 (2014)].

Uncertainty relations^1^ are of profound significance in quantum mechanics and quantum information theory. Various important applications of uncertainty relations have been discovered such as entanglement detection^2^, steering inequalities^3^ and quantum cryptography^4^,^5^,^6^. The well-known form of the Heisenberg’s uncertainty relations, given by Robertson^7^, says that the standard deviations of the observables Δ*A* and Δ*B* satisfy the following inequality,


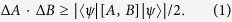


As a consequence of the uncertainty relations, it is impossible to determine the exact values of the two incompatible observables simultaneously. However, the lower bound in the above uncertainty inequality may become trivial if the measured state |*ψ*〉 belongs to the nullspace of the commutator [*A, B*].

In fact, the uncertainty relations provide a limitation on how much information one can obtain by measuring a physical system, and can be characterized in terms of the probability distributions of the measurement outcomes. In order to overcome the drawback in the product form of variance base uncertainty relations, Deutsch^8^ introduced the entropic uncertainty relations, which were later improved by Maassen and Uffink^9^: *H*(*A*) + *H*(*B*) ≥ −2 log *c*(*A, B*), where *H* is the Shannon entropy, 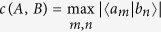 is maximum overlap between the basis elements {|*a*_*m*_〉} and {|*b*_*n*_〉} of the eigenbases of *A* and *B*, respectively. Recently, the Maassen-Uffink bound has been surprisingly improved by Coles and Piani^10^, Rudnicki, Pucha
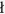
a and 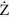
yczkowski^11^, for a review on entopic uncertainty relations see refs 12 and 13.

Friedland, Gheorghiu and Gour^14^ proposed a new concept called “universal uncertainty relations” which are not limited to considering only the well-known entropic functions such as Shannon entropy, Renyi entropy and Tsallis entropy, but also any nonnegative Schur-concave functions. On the other hand, Pucha
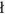
a, Rudnicki and 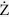
yczkowski^15^ independently used majorization technique to establish entropic uncertainty relations similar to “universal uncertainty relations”. Let  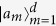 and 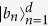 be orthonormal bases of a *d*-dimensional Hilbert space *H*. Denote by *p*_*m*_(*ρ*) = 〈*a*_*m*_|*ρ*|*a*_*m*_〉 and *q*_*n*_(*ρ*) = 〈*b*_*n*_|*ρ*|*b*_*n*_〉 the probability distributions obtained by measuring the state *ρ* with respect to these bases, which constitute two probability vectors **p**(*ρ*) = (*p*_1_, *p*_2_, …, *p*_*d*_) and **q**(*ρ*) = (*q*_1_, *q*_2_, …, *q*_*d*_), respectively. It has been shown that the tensor product of the two probability vectors **p**(*ρ*) and **q**(*ρ*) is majored by a vector *ω* independent from the state *ρ*,


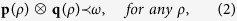


where “
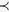
” stands for “majorization”: 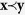 in *R*^*d*^ if 
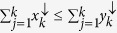
 for all 1 ≤ *k* ≤ *d* − 1 and 
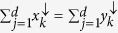
. The down-arrow vector **x**^↓^ denotes that the components of **x** are rearranged in descending order, 
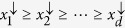
. The *d*^2^-dimensional vector *ω* is given by


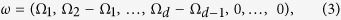


where


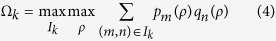


with *I*_*k*_ ⊂ [*d*] × [*d*] being a subset of *k* distinct pairs of indices (*m, n*) and [*d*] is the set of the natural numbers from 1 to *d*. The outer maximum is over all subsets *I*_*k*_ with cardinality *k* and the inner maximum runs over all density matrices.

Equation (2) is called a universal uncertainty relation, as for any uncertainty measure Φ, a nonnegative Schur-concave function, one has that


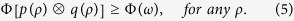


The universal uncertainty relation (UUR) (2) generates infinitely many uncertainty relations, for each Φ, in which the right hand side provides a single lower bound.

In relation (2), the state independent vector *ω* decided by Ω_*k*_ in Eq. (4) is too hard to evaluate explicitly in general, as it is involved with a highly nontrivial optimization problem. For this reason, only an approximation 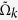 of Ω_*k*_ has been presented^14^,^15^ to construct a weaker majorization vector 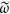. Naturally, how to find a stronger approximation than previous works becomes an interesting open question.

## Results

We first introduce a scheme called “joint probability distribution diagram” (JPDD) to consider the optimization problem involved in calculating Ω_*k*_. Next, we present a stronger approximation by proposing an analytical formula for Ω_*k*_. To facilitate presentation, we denote our stronger approximation as Ω_*k*_ without ambiguity. All uncertainty relations considered in the paper will be in the absence of quantum side information.

**Figure 1:**
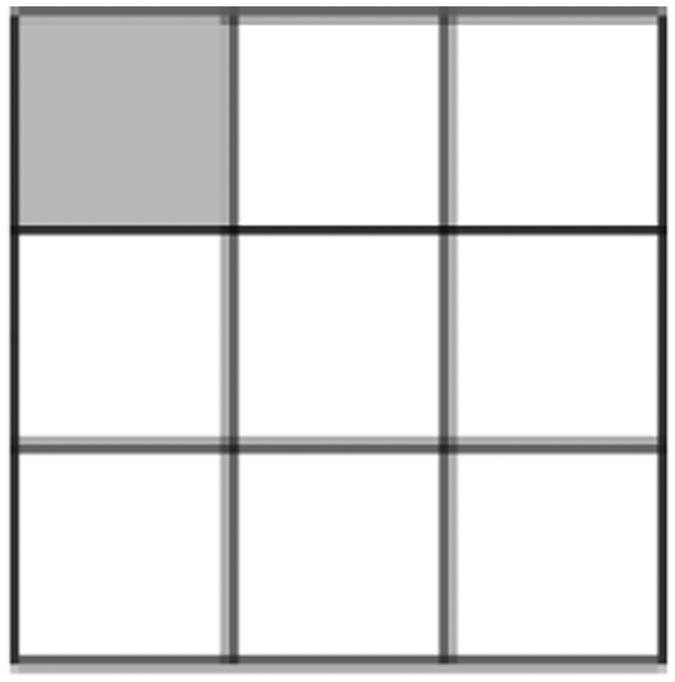
The left-top shadow of the block box specifies the entry *p*_1_*q*_1_.

To construct the joint probability distribution diagram, we associate each summand *p*_*i*_(*ρ*)*q*_*j*_(*ρ*) in Ω_*k*_ to a box located at the position (*i, j*). Then the summation in Ω_*k*_ corresponds to certain region of boxes (or rather lattice points) in the first quadrant. We configure the region in a combinatorial way. Suppose that *p*_1_ ≥ *p*_2_ ≥ ··· ≥ *p*_*d*_, *q*_1_ ≥ *q*_2_ ≥ ··· ≥ *q*_*d*_. Consider the following *d* × *d*-matrix


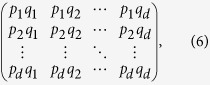


where the entries descend along the rows and columns by assumption. Now, we use a box □ to represent an entry of the matrix. A *shadow* or *grey box* in the JPDD means the corresponding entry in the matrix. For example, the top left shadow of the block box specifies the entry *p*_1_*q*_1_, see Fig. 1. Thus the region corresponding to the summation in 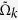 will be a special region of the rectangular matrix.

Our scheme, JPDD, provides a combinatorial method to compute the special region with respect to the Ω_*k*_. First, it is easy to see that the top upper left box in JPDD is the maximal element, i.e. Ω_1_, since *p*_1_*q*_1_ − *p*_*i*_*q*_*j*_ ≥ *p*_*i*_*q*_1_ − *p*_*i*_*q*_*j*_ = *p*_*i*_(*q*_1_ − *q*_*j*_) ≥ 0. The main idea is that each exact solution of Ω_*k*_ corresponds to a particular region in this matrix.

Suppose that the *k*-th region is found, i.e. Ω_*k*_ is obtained, then the next (*k* + 1)-th region is obtained from the *k*-th region by adding a special box, which must be “connected” with certain boundary of the *k*-th region. This iterative procedure enables us to compute all Ω_*k*_. Before proving the statement rigorously, we first introduce some terminologies.

**Figure 2:**
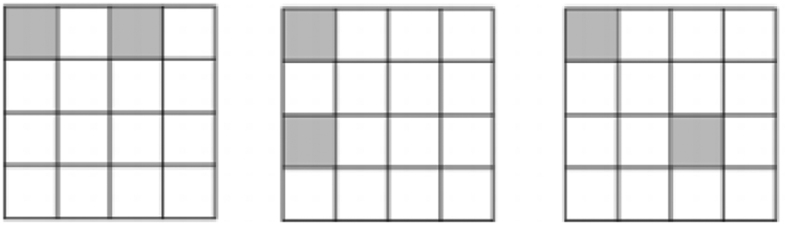
Different boxes.

[Definition 1] (Different boxes) Two boxes (matrix elements) *p*_*i*_*q*_*j*_ and *p*_*k*_*q*_*l*_ are said to be *different* if they occupy different positions in JPDD, namely, *i* ≠ *k* or *j* ≠ *l*. The Fig. 2 shows three examples of different boxes. Note that it may happen that even if the numerical values of *p*_*i*_*q*_*j*_ and *p*_*k*_*q*_*l*_ are the same, but graphically they are treated as different boxes. “different” and “same” do not imply their quantitative relation. For example, *p*_1_*q*_1_ may equal to *p*_1_*q*_3_ in general.

**Figure 3:**
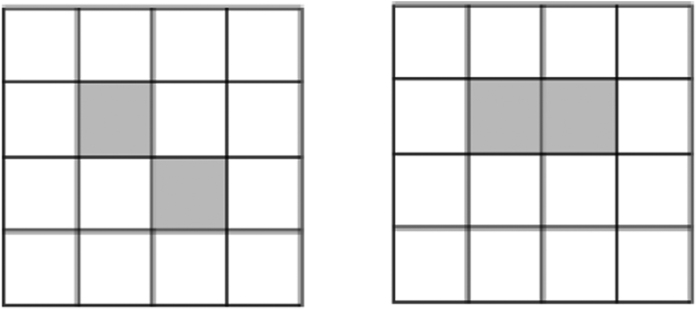
Connectedness.

[Definition 2] (Connectedness). Two boxes *p*_*i*_*q*_*j*_ and *p*_*k*_*q*_*l*_ in JPDD are *connected* if there does not exist any box *p*_*m*_*q*_*n*_, different from both *p*_*i*_*q*_*j*_ and *p*_*k*_*q*_*l*_, such that min{*p*_*i*_*q*_*j*_, *p*_*k*_*q*_*l*_} < *p*_*m*_*q*_*n*_ < max{*p*_*i*_*q*_*j*_, *p*_*k*_*q*_*l*_}. For example, different boxes *p*_*i*_*q*_*j*_ and *p*_*k*_*q*_*l*_ are connected if *p*_*i*_*q*_*j*_ = *p*_*k*_*q*_*l*_. For *d* = 4, if *p*_1_ > *p*_2_ > *p*_3_ > *p*_4_, *q*_1_ > *q*_2_ > *q*_3_ > *q*_4_, *p*_*i*_*q*_*j*_ ≠ *p*_*k*_*q*_*l*_, *p*_*m*_*q*_*n*_ ≠ *p*_*i*_*q*_*j*_, *p*_*m*_*q*_*n*_ ≠ *p*_*k*_*q*_*l*_, for any *i, j, k, l* ∈ {1, 2, 3, 4}, then *p*_2_*q*_2_ and *p*_3_*q*_3_ are not connected while *p*_2_*q*_3_ and *p*_2_*q*_2_ are connected, see Fig. 3.

[Definition 3] (Connected region). A set of different boxes is called a *region*, denoted by 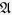. A region 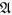 is *connected* if 
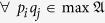
, then either 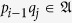 or 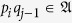, where 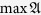 is the maximal value of all the elements in 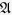. Note that the region of boxes corresponding to a Ω_*k*_ must contain the top-left element *p*_1_*q*_1_ in JPDD as its largest element.

**Figure 4:**
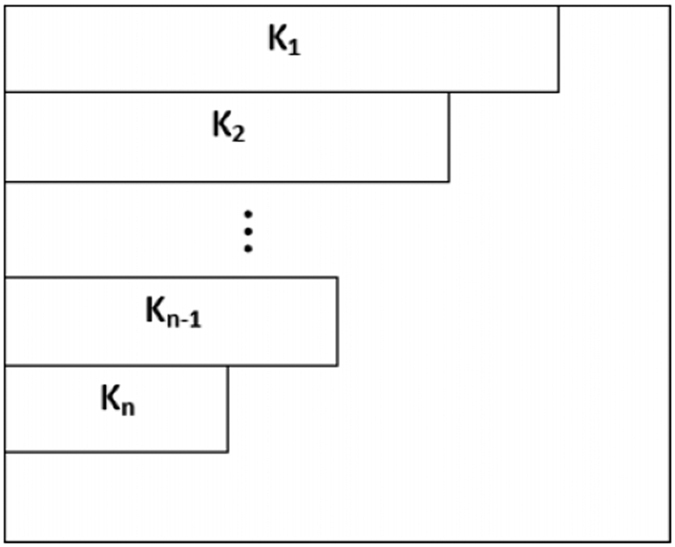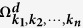 related joint probability distribution diagram.

For any probability vector **p**(*ρ*) on a *d*-dimensional Hilbert space with *p*_*i*_ = 〈*a*_*i*_|*ρ*|*a*_*i*_〉, let 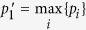, 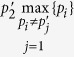,  ··· , 
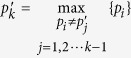
. Similarly, 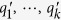 are defined similarly for another probability vector **q**(*ρ*) on the same Hilbert space. For any sequence *k*_1_,  ··· , *k*_*n*_, 1 ≤ *n* ≤ *d*, we define 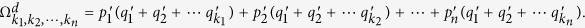 In particular, if *p*_1_ ≥ ··· ≥ *p*_*d*_, *q*_1_ ≥ ··· ≥ *q*_*d*_, then 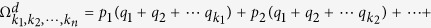

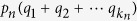
, which can be configured by the Fig. 4. In a JPDD when the first *k* boxes are chosen, the next (maximal) (*k* + 1)-th box must appear at the top left corner in the unoccupied region, we give this as follow lemma:

[Lemma]: The maximal *k* boxes for Ω_*k*_ in the JPDD can be selected to form a connected region. ■

Lemma gives a way to get Ω_*k*+1_ from Ω_*k*_ in a JPDD. As an example, we show how to get Ω_3_ from Ω_2_. Set 
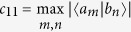
, 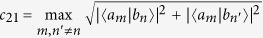, 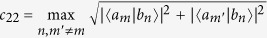. If *c*_21_ ≥ *c*_22_, then 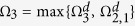. If *c*_21_ ≤ *c*_22_, then 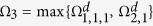. That is, if 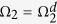, then 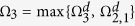. If 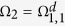, then 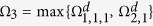. Namely, 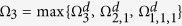. Thus Ω_3_ is determined by Ω_2_.

In general, 
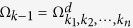
, subjecting to 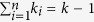. By Lemma it follows that


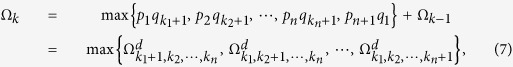


which gives an iterative formula of Ω_*k*_ in terms of 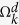
’s.

**Figure 5:**
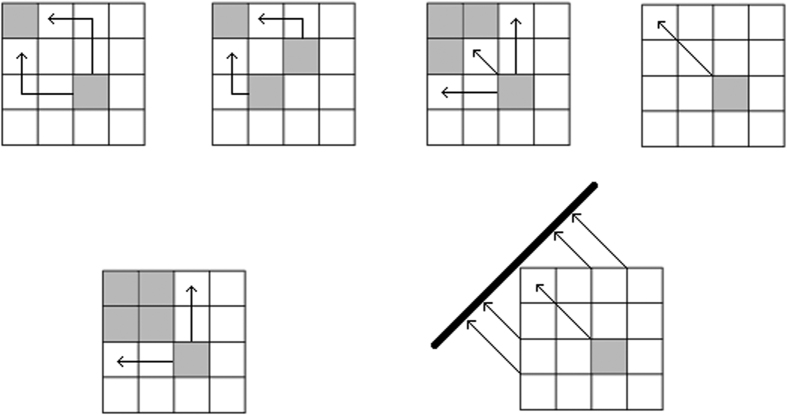
The (k + 1)-th box is fixed by Ω_*k*_. The arrows show the position of the (k + 1)-th box.

**Figure 6:**
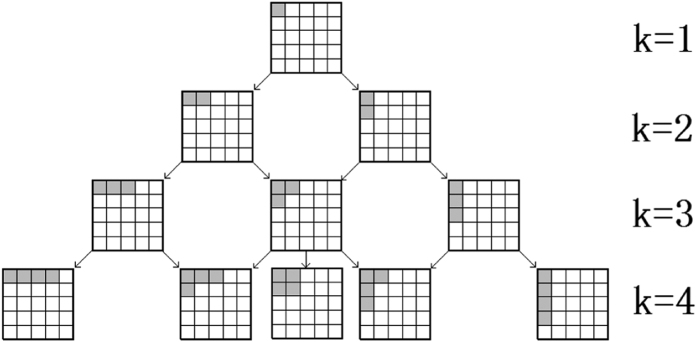
The tree diagrams to get the JPDDs in the *k*-th row by adding one shadow box to the JPDDs in the *k* − 1 row.

We list in Figs 5 and 6 all the possible Ω_*k*_ for *k* = 1, 2 ,..., 4. The above example to get Ω_3_ from Ω_2_ corresponds to move from the second row to the third. Now we are ready to show the main result.

[Theorem] The quantities Ω_*k*_ are given by


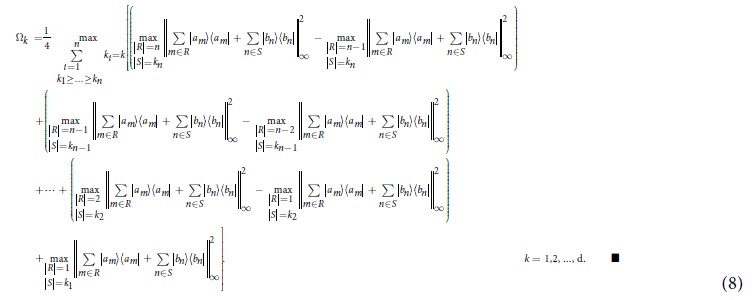


The solution given in Eq. (8) can be explained as follows. First, for *k* = 1, 2, they are solved simply as


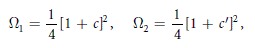


where 
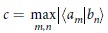
, 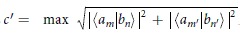, the maximum is taken over all indices *m* ≠ *m*′, *n* ≠ *n*′, and over all *n* = *n*′,*m* ≠ *m*′. Then, for *k* = 3 in JPDD, 
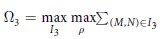

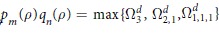
 Furthermore, 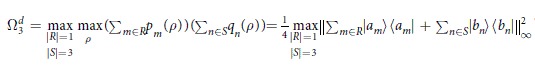
and 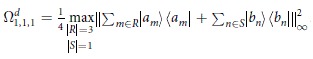. Since 
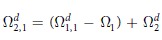
, we get 
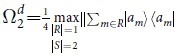

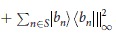
 and 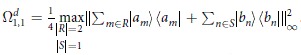 We have shown how to calculate Ω_1_, Ω_2_ and Ω_3_. For the cases *k* ≥ 4, interested readers can calculate Ω_*k*_ using a similar method and we sketch the details in the Methods.

The above theory enables us to formulate a series of Ω_*k*_, based on all quantities we obtain a tighter majorization vector *ω*. Note that our method is valid when all the maximums are taken over the same quantum state, otherwise our bounds will fail to hold. Even so, our results can outperform *B*_*Maj*2_^11^ to some extent.

Our results enable us to strengthen the bounds on the sum of two Shannon entropies by **B**_**JPDD**_ = *H*(*ω*), where *ω* is given by the improved Ω_*k*_ in Eq. (8) and *H* is the Shannon entropy. To see this phenomenon, let us first consider a 4-dimensional system with incompatible observables 
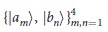



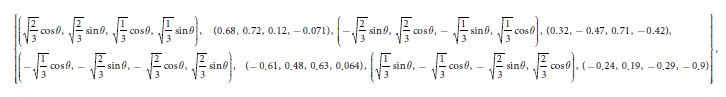


and the unitary transformation *U (U*_*mn*_ = 〈*a*_*m*_|*b*_*n*_〉) between them


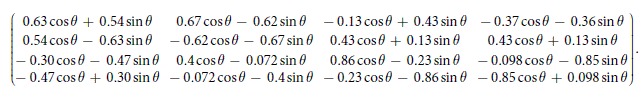


**Figure 7:**
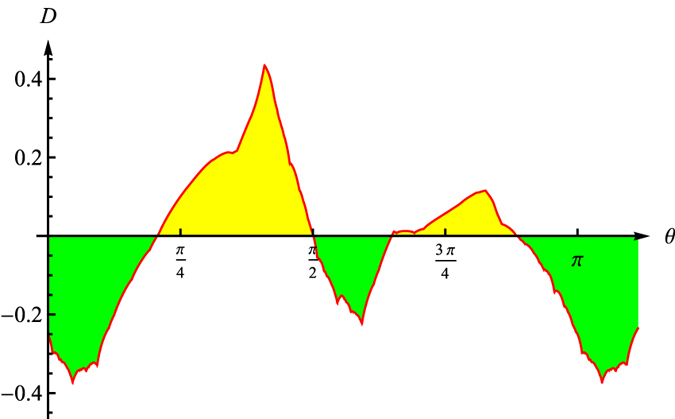
Difference between *B*_*JPDD*_ and *B*_*Maj*2_.

In Fig. 7, we plot the difference between **B**_**JPDD**_ and *B*_*Maj*2_^11^, i.e. *B*_*JPDD*_ − *B*_*Maj*2_ (the red line). Clearly, our bound *B*_*JPDD*_ is tighter than *B*_*Maj*2_ to some extent. Note that, the entropies are defined with base 2 in general. But in our figures, in order to make them more readable, we take the natural logarithm instead.

**Figure 8:**
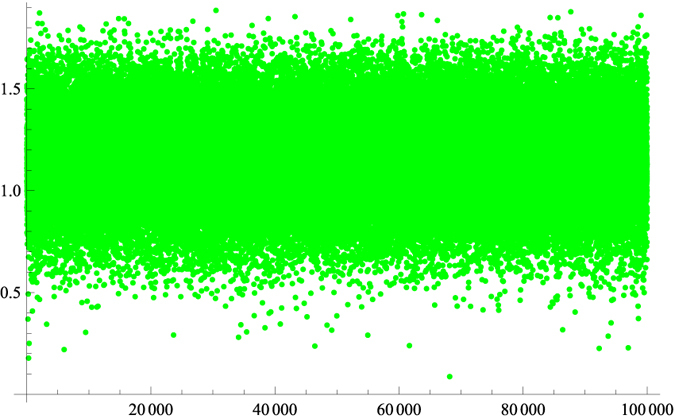
The vertical coordinate is *G*(*c*) − *F*(*c*). The horizontal coordinate is for random runs. It can be seen that our bound outperforms the bound^16^ 100% of the time, while a bound given by Friedland^14^ outperforms the bound^16^ around 90% of the time.

To appreciate the stronger vector *ω* in the improved UUR, we can consider Shannon entropy in the uncertainty relations to obtain a tighter bound than the previous work^16^. Namely if we Φ in Eq. (5) as the Shannon entropy *H*, then we have


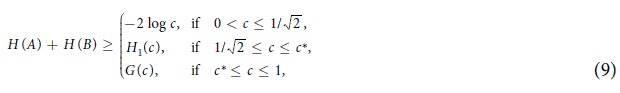


where c^*^ ≈ 0.834, the bound *H*_1_(*c*) is the same as the one given in Vincente and Ruiz’s work^16^, and *G*(*c*) = *H*(*ω*) with *ω* given by our formula Eq. (3). The bound *G*(*c*) outperforms the Vincente and Ruiz’s bound *F*(*c*)^16^ in the interval [c^*^, 1]. For further details, see Fig. 8.

## Conclusion

In conclusion, we have presented a method called joint probability distribution diagram to strengthen the bounds for the universal uncertainty relations. As an example, we consider the bounds on the sum of the Shannon entropies. As the universal uncertainty relations capture the essence of uncertainty in quantum theory, it is unnecessary to quantify them by particular measures of uncertainty such as Shannon or Renyi entropies. Our results give a way to resolve some important cases in this direction, and is shown to offer a better bound for any uncertainty relations given by the nonnegative Schur-concave functions. Furthermore, how to extend this method to the case of multiple measurements are interesting, which requires further studies.

## Methods

### Proof of the Lamma

The case of *k* = 1 is obvious since the maximal element is *p*_1_*q*_1_. Assume that the statement holds for the case of *k* − 1: 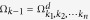 is connected with *k*_1_ > … > *k*_*n*_ > 0. Suppose on the contrary that the next maximum Ω_*k*_ = Ω_*k* − 1_ + *p*_*i*_*q*_*j*_ is not connected. Then there are two possibilities: (i) *i* > *n* or *j* > *k*_1_, thus we can replace *p*_1_*q*_*j*_ by *p*_*i*_*q*_1_ or *p*_1_*q*_*j*_ and move further to *p*_*n*+1_*q*_1_ or 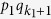 to get a possible bigger value for Ω_*k*_. (ii) *i* ≤ *n* and *j* > *k*_*i*_, in this case we can also replace the box *p*_*i*_*q*_*j*_ by a connected box *p*_*i*′_*q*_*j*′_ to the region of Ω_*k* − 1_ by sliding it leftward or upward. Hence the statement is true by induction. ■

### Proof of the Theorem

To calculate 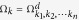, *k*_1_ ≥ … ≥ *k*_*n*_ and *k*_1_ + ··· + *k*_*n*_ = *k*, we note that


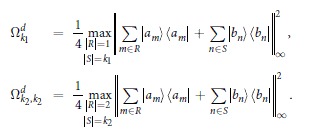


where *R* and *S* are subsets of distinct indices from [d], |*R*| is the cardinality of *R*, and ||·||_∞_ is the infinity operator norm which coincides with the maximum eigenvalue of the positive operator. For a given *k*, there exist sets of *k*_1_ ≥ … ≥ *k*_*n*_ such that 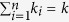 for some *n*. For any such given *k*_1_ ≥ … ≥ *k*_*n*_, the quantity in [.] in Eq. (8) can be calculated. The outer max picks up the largest quantity for all such possible *k*_1_, …, *k*_*n*_. Then 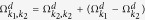 and 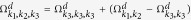. Continuing in this way we have


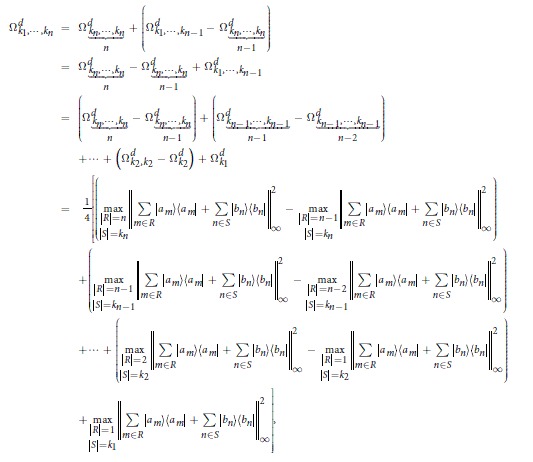


which gives the improved values of 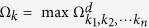, where max is taken over 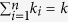 with *k*_1_ ≥ … ≥ *k*_*n*_, and hence *ω* = (Ω_1_, Ω_2_ − Ω_1_, …, Ω_*d*_ − Ω_*d*−1_, 0, …, 0) for any *ρ*. ■

## Additional Information

**How to cite this article**: Li, T. *et al*. Optimal Universal Uncertainty Relations. *Sci. Rep.* **6**, 35735; doi: 10.1038/srep35735 (2016).

## References

[b1] Heisenberg W. Über den anschaulichen Inhalt der quantentheoretischen Kinematik und Mechanik. Zeitschrift für Physik 43(3**–4**), 172–198 (1927).

[b2] Gühne O. Characterizing entanglement via uncertainty relations. Phys. Rev. Lett. 92(11), 117903 (2004). 15089173 10.1103/PhysRevLett.92.117903

[b3] Schneeloch J., Broadbent C. J. & Howell J. C. Cryptography from Noisy Storage. Phys. Lett. A 378, 766 (2014).

[b4] Koashi M. Simple security proof of quantum key distribution based on complementarity. New J. Phys. 11(4), 045018 (2009).

[b5] Renes J. M. & Boileau J. C. Physical underpinnings of privacy. Phys. Rev. A 78(3), 032335 (2008).

[b6] Tomamichel M., Lim C. C. W. & Gisin N. . Tight finite-key analysis for quantum cryptography. Nature commun. 3, 634 (2012). 22252558 10.1038/ncomms1631 PMC3274703

[b7] Robertson H. P. The uncertainty principle. Phys. Rev. 34(1), 163 (1929).

[b8] Deutsch D. Uncertainty in quantum measurements. Phys. Rev. Lett. 50(9), 631 (1983).

[b9] Maassen H. & Uffink J. B. M. Generalized entropic uncertainty relations. Phys. Rev. Lett. 60(12), 1103 (1988). 10037942 10.1103/PhysRevLett.60.1103

[b10] Coles P. J. & Piani M. Improved entropic uncertainty relations and information exclusion relations. Phys. Rev. A 89(2), 022112 (2014).

[b11] Rudnicki Ł. Puchała Z. & Życzkowski K. Strong majorization entropic uncertainty relations. Phys. Rev. A 89(5), 052115 (2014).

[b12] Wehner S. & Winter A. Entropic uncertainty relations – a survey. New J. Phys. 12(2), 025009 (2010).

[b13] Coles P. J., Berta M. & Tomamichel M. . Entropic Uncertainty Relations and their Applications. arXiv, 1511.04857 (2015)

[b14] Friedland S., Gheorghiu V. & Gour G. Universal uncertainty relations. Phys. Rev. Lett. 111(23), 230401 (2013). 24476234 10.1103/PhysRevLett.111.230401

[b15] Puchała Z., Rudnicki Ł. & Życzkowski K. Majorization entropic uncertainty relations. J. Phys. A: Math. and Theor. 46(27), 272002 (2013).

[b16] de Vicente J. I. & Sänchez-Ruiz J. Improved bounds on entropic uncertainty relations. Phys. Rev. A 77(4), 042110 (2008).

